# A Point Mutation in the Gene for Asparagine-Linked Glycosylation 10B (*Alg10b*) Causes Nonsyndromic Hearing Impairment in Mice (*Mus musculus*)

**DOI:** 10.1371/journal.pone.0080408

**Published:** 2013-11-26

**Authors:** Frank J. Probst, Rebecca R. Corrigan, Daniela del Gaudio, Andrew P. Salinger, Isabel Lorenzo, Simon S. Gao, Ilene Chiu, Anping Xia, John S. Oghalai, Monica J. Justice

**Affiliations:** 1 Department of Molecular and Human Genetics, Baylor College of Medicine, Houston, Texas, United States of America; 2 Genetically Engineered Mouse Shared Resource, Baylor College of Medicine, Houston, Texas, United States of America; 3 Department of Bioengineering, Rice University, Houston, Texas, United States of America; 4 Department of Otolaryngology-Head and Neck Surgery, Baylor College of Medicine, Houston, Texas, United States of America; 5 Department of Otolaryngology-Head and Neck Surgery, Stanford University, Stanford, California, United States of America; IGBMC/ICS, France

## Abstract

The study of mouse hearing impairment mutants has led to the identification of a number of human hearing impairment genes and has greatly furthered our understanding of the physiology of hearing. The novel mouse mutant neurological/sensory 5 (*nse5*) demonstrates a significantly reduced or absent startle response to sound and is therefore a potential murine model of human hearing impairment. Genetic analysis of 500 intercross progeny localized the mutant locus to a 524 kilobase (kb) interval on mouse chromosome 15. A missense mutation in a highly-conserved amino acid was found in the asparagine-linked glycosylation 10B gene (*Alg10b*), which is within the critical interval for the *nse5* mutation. A 20.4 kb transgene containing a wildtype copy of the *Alg10b* gene rescued the mutant phenotype in *nse5*/*nse5* homozygous animals, confirming that the mutation in *Alg10b* is responsible for the *nse5*/*nse5* mutant phenotype. Homozygous *nse5*/*nse5* mutants had abnormal auditory brainstem responses (ABRs), distortion product otoacoustic emissions (DPOAEs), and cochlear microphonics (CMs). Endocochlear potentials (EPs), on the other hand, were normal. ABRs and DPOAEs also confirmed the rescue of the mutant *nse5*/*nse5* phenotype by the wildtype *Alg10b* transgene. These results suggested a defect in the outer hair cells of mutant animals, which was confirmed by histologic analysis. This is the first report of mutation in a gene involved in the asparagine (N)-linked glycosylation pathway causing nonsyndromic hearing impairment, and it suggests that the hearing apparatus, and the outer hair cells in particular, are exquisitely sensitive to perturbations of the N-linked glycosylation pathway.

## Introduction

Congenital hearing impairment in the human (*Homo sapiens*) population has a newborn prevalence of 0.1–0.3% in live births in the United States [1]. Previously, about half of these cases in the developed world were believed to be genetic, while the remaining half were thought to be entirely due to other causes, such as congenital infections [2]. More recent research has suggested that genetic factors may play a role in susceptibility to hearing impairment even when the primary cause is non-genetic, such as congenital cytomegalovirus (CMV) infection [3].

Mutations in the gene for connexin-26 (*GJB2*, OMIM *121011) are the most common genetic causes of congenital hearing impairment (OMIM #220290) and account for up to half of the genetic cases in some populations. However, more than 120 additional human hearing loss loci have been reported, and the responsible genes have been identified for only about half of these [2], [4], [5].

Current treatment options for genetic hearing impairment are largely limited to hearing aids or cochlear implants [6]. Animal studies have demonstrated that adenoviral vectors can be introduced into the inner ear to regenerate lost sensorineural cells [7] and to transfer gene expression constructs to cochlear tissue [8]. An adeno-associated virus type 1 vector has recently been used to restore hearing to congenitally-deaf mice with vesicular glutamate transporter-3 deficiency (official gene symbol *Slc17a8*, MGI: 3039629) [9]. In addition, a single systemic dose of an antisense oligonucleotide (ASO) in neonatal mice with an *Ush1c* (MGI: 1919338) mutation has recently been shown to rescue the mutant phenotype of deafness and blindness in these mice [10]. These studies suggest that gene-specific therapies are a viable strategy for treating genetic hearing impairment. However, before therapy can be performed, the underlying genetic defect in each individual case must be identified. Thus, a search for the remaining unknown genes responsible for hearing impairment is warranted.

Mouse mutants have long been useful tools for the identification of human hearing loss genes and for the study of the physiology of hearing [11]–[14]. The study of human hearing loss genes is complicated by locus heterogeneity, genetic background effects, small pedigree size, selective mating, and difficulty in performing physiological and histological studies. Each of these issues can be avoided in the mouse. Furthermore, mice can be intentionally mutagenized and screened for hearing impairment in order to generate novel mouse models of human hearing deficits [15]. Indeed, chemical mutagenesis has successfully produced a number of mouse mutants with mutations in the gene for chromodomain helicase DNA-binding protein-7 (*Chd7*, MGI: 2444748) [16], which now serve as mouse models for CHARGE syndrome (OMIM #214800; *CHD7*, OMIM *608892), a form of syndromic hearing impairment [17].

During the course of a germline mutagenesis screen, the neurological/sensory 5 (*nse5*, MGI: 2671810) mouse mutation arose in the progeny of a C57BL/6J male mouse that had been treated with the chemical supermutagen *N*-ethyl-*N*-nitrosourea (ENU). Homozygous *nse5*/*nse5* mutant animals have a severely reduced or absent startle response to sound at weaning (3 weeks of age) and virtually no response to sound at 6 weeks of age. While many mouse hearing impairment mutants also have associated head-tossing or circling behaviors (presumably secondary to associated vestibular defects), *nse5* homozygotes behave normally, implying that the mutation affects the cochlea but spares the vestibular apparatus. Mutant animals are otherwise completely normal on examination and have normal complete blood counts and blood chemistries, suggesting that the hearing impairment is nonsyndromic. A genome scan previously mapped the responsible locus to mouse chromosome 15 in a region where there were no reported hearing impairment genes, indicating that *nse5* represents a novel locus for hearing impairment [18].

We report here the fine-mapping of the *nse5* mutation, the identification of a leucine-to-serine mutation in the *Alg10b* gene in mutant animals, rescue of the mutant phenotype with a wildtype *Alg10b* transgene, and physiologic and histologic analysis of wildtype, mutant, and transgenic animals. The ALG10B protein is involved in the process of asparagine (N)-linked glycosylation. This is the first report of a mutation in this pathway resulting in nonsyndromic hearing impairment in either the mouse or in humans, and it suggests that this pathway is much more important than previously known in the establishment and maintenance of normal hearing.

## Results

### The *nse5* Mutation Maps to a 524 Kilobase (kb) Region of Mouse Chromosome 15

**Figure 1:**
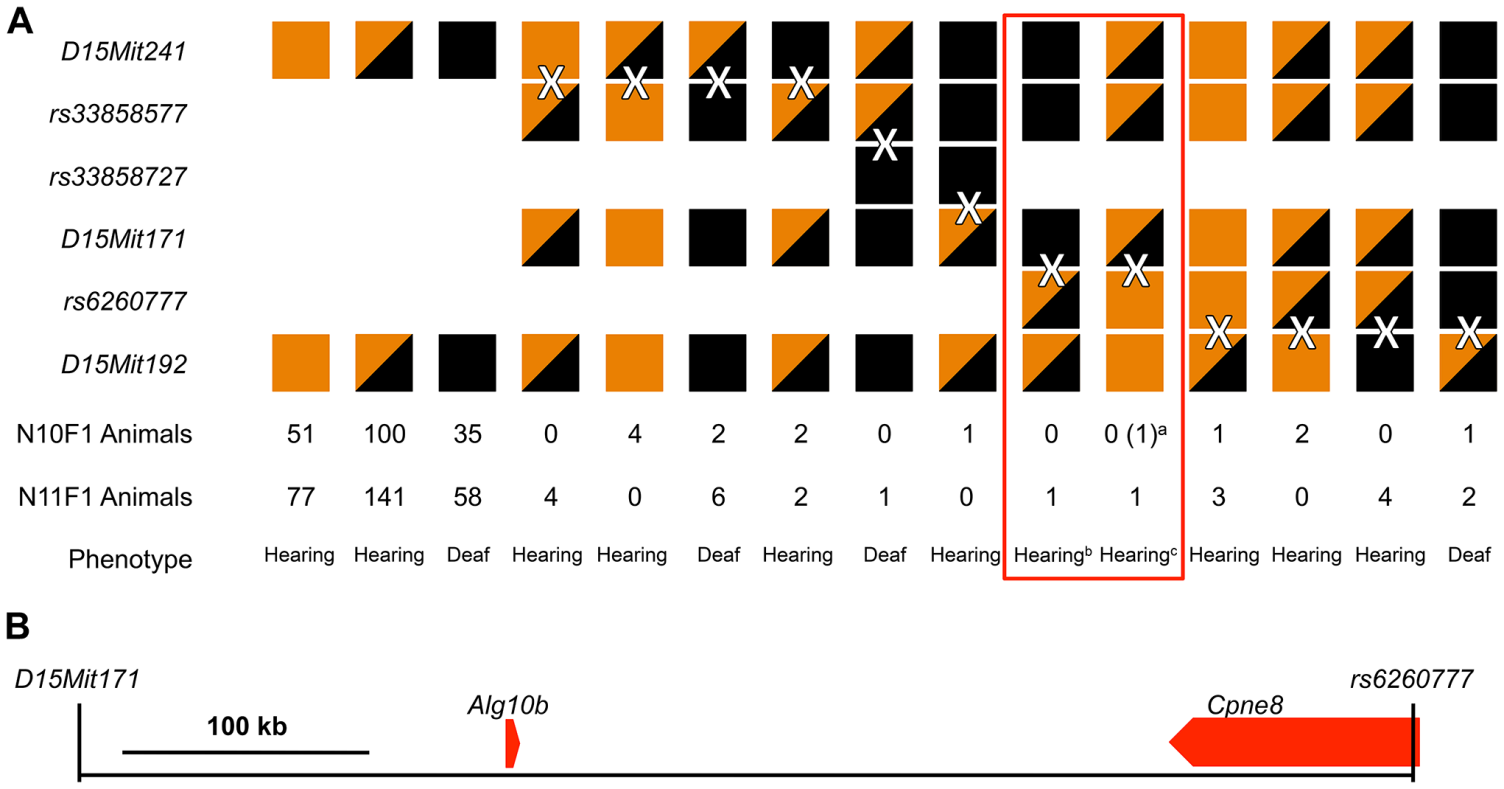
Genetic mapping of the neurological/sensory 5 (*nse5*) mutation. (A) The *nse5* mouse mutation was localized to a genomic interval using traditional intercrossing of N10 and N11 animals. All animals were genotyped at the *D15Mit241* and *D15Mit192* loci. Animals with discordant genotypes between these two loci were considered recombinants and genotyped at additional loci, as shown, to localize the recombination breakpoints (shown as “X”es). A brown box indicates homozygosity for the 129S6/SvEvTac allele, a black box indicates homozygosity for the C57BL/6J allele, and half-brown/half-black boxes indicate heterozygosity for the two alleles. The number of each N10F1 and N11F1 animals with each haplotype is shown, as are the phenotypes of the mice with that particular haplotype, with “Hearing” indicating a clear startle response on click-box testing, and “Deaf” indicating an absent startle response. As shown by the red box, this analysis allowed us to localize the *nse5* mutation to region of mouse chromosome 15 between *D15Mit171* and *rs6260777*. (B) This region is 524 kb in size and contains only two RefSeq genes, *Alg10b* and the 3′ end of the *Cpne8* gene. ^a^An N10 animal is included here (shown in parentheses), as this animal was found to have a critical recombination breakpoint during our routine genotyping of N10 and N11 animals. ^b^This animal was classified as “Hearing”, suggesting that its genotype was +/*nse5* at the *nse5* locus. When backcrossed to an *nse5*/*nse5* mouse, five progeny that had inherited the recombinant chromosome were also phenotyped as “Hearing”, confirming the +/*nse5* genotype and indicating that the *nse5* locus is distal to *D15Mit171*. ^c^These two mice were both classified as “Hearing”. However, their genotype at the *nse5* locus could only be inferred as +/?, since they could be either +/+ or +/*nse5*. Both animals were therefore backcrossed to *nse5*/*nse5*. Unfortunately, the N11F1 animal died prior to producing any progeny. Phenotyping of five progeny from the N10 animal that had inherited the recombinant chromosome revealed that all were “Deaf”, indicating that the N10 animal was +/*nse5* at the *nse5* locus and placing the *nse5* locus proximal to *rs6260777*.

Analysis of 200 N10F1 and 300 N11F1 mouse progeny (and one additional N10 mouse that was found to have a critical recombination breakpoint) localized the *nse5* locus to a nonrecombinant interval between the markers *D15Mit171* and *rs6260777* on mouse chromosome 15 (Figure 1A). One nonrecombinant animal was initially scored as “Hearing” but was reclassified as “Deaf” when re-examined one week later. Another animal with a recombination between *D15Mit241* and *rs33858577* had a persistent but very subtle startle response on click-box testing. This animal was ultimately classified as “Deaf”. These two animals demonstrate the variable expressivity of the phenotype, which is a common feature of ENU-induced mutations. Given this finding, the two animals carrying the key recombinants were both backcrossed to *nse5*/*nse5* animals, and at least five animals that inherited the recombinant chromosome were phenotyped, in order to guard against mistyping a critical animal.

Review of the published genomic sequence of this region with the UCSC Genome Browser revealed that it is 524 kb in length and contains only two RefSeq genes, *Alg10b* and the 3′ end of the *Cpne8* gene (Figure 1B) [19], [20].

### A Missense Mutation in *Alg10b* Is Present in *nse5* Mutant Mice

**Figure 2:**
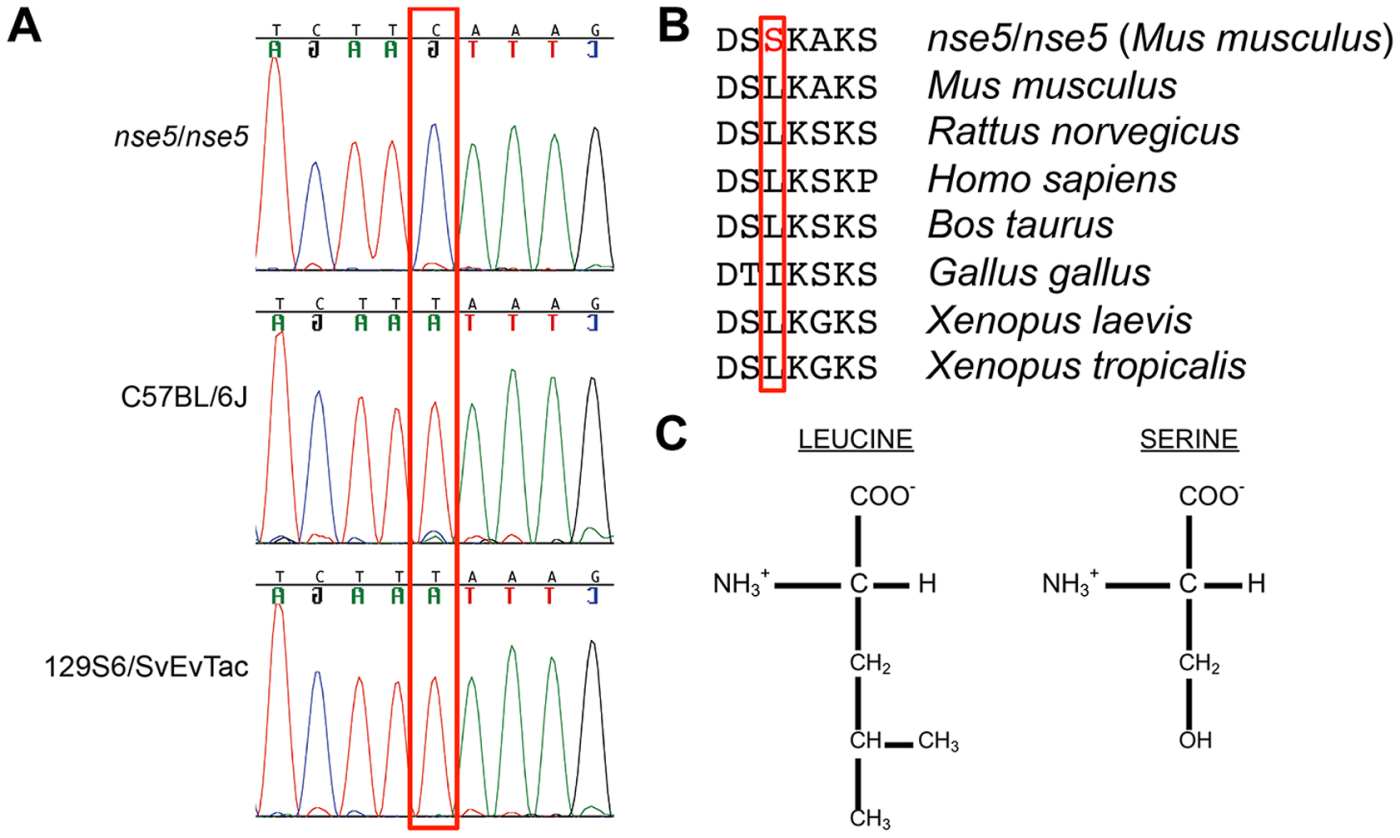
Identification of a leucine-to-serine missense mutation in a highly-conserved region of *Alg10b*. (A) Chromatograms from *nse5*/*nse5*, C57BL/6J, and 129S6/SvEvTac DNA are shown, demonstrating a novel C>T transition in *nse5*/*nse5* DNA, boxed in red. (B) This change converts a highly conserved leucine into a serine residue. (C) Leucine is a bulky, nonpolar amino acid, while serine is a small, polar amino acid. (Reference sequences for (B): *Mus musculus*, NP_001028613.1; *Rattus norvegicus*, NP_620801.1; *Homo sapiens*, NP_001013642.1; *Bos taurus*, NP_001069657.1; *Gallus gallus*, NP_001026565.1; *Xenopus laevis*, NP_001084985.1; and *Xenopus tropicalis*, NP_001016192.2.)

Amplification and sequencing of the three coding exons and the 5′-untranslated region (UTR) of the gene for asparagine-linked glycosylation 10B (*Alg10b*) from an *nse5*/*nse5* homozygous mutant animal revealed a single point mutation in this gene that was not present in the reference sequence. Analysis of DNA from both C57BL/6J and 129S6/SvEvTac inbred animals revealed that this mutation is not present in either strain (Figure 2A). The mutation converts a highly-conserved leucine residue to a serine residue in mutant animals (p.Leu389Ser, based on reference sequence NP_001028613.1, conservation shown in Figure 2B). Leucine is a bulky, relatively non-polar amino acid, while serine is small and polar, due its hydroxyl group (Figure 2C). Analysis of this protein change with Poly-Phen2 v2.2.2r398 predicted that this change is probably damaging (score: 0.998; sensitivity: 0.27; specificity 0.99) using the HumDiv model and also probably damaging (score: 0.947; sensitivity 0.65; specificity 0.92) using the HumVar model [21].

### A 20.4 kb Genomic Transgene Containing the Wildtype *Alg10b* Gene Rescues the Mutant Phenotype

**Figure 3:**
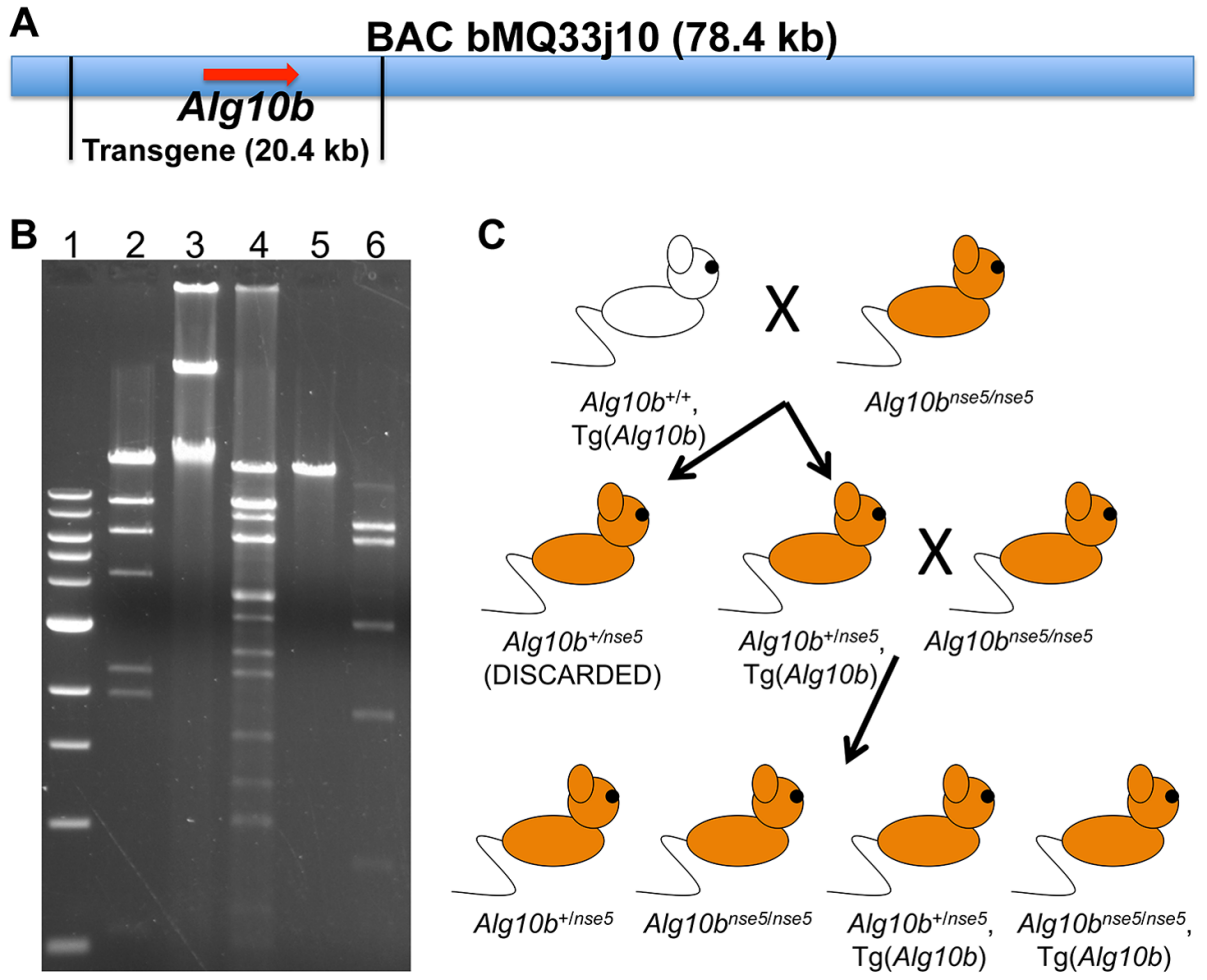
A wildtype *Alg10b* transgene can rescue the *nse5* mutant phenotype. (A) A schematic showing the 78.4 kb insert of BAC bMQ33j10, which contains only the *Alg10b* gene and no other genes. The desired 20.4 kb transgene containing the *Alg10b* gene is shown. (B) Gel electrophoresis demonstrating the isolation of a 20.4 kilobase (kb) DNA fragment from the BAC. Lanes 1 and 2, 1 kb DNA ladder and Lambda DNA-*Hin*dIII digest ladder (New England Biolabs, Ipswich, MA), with the highest bands at 10.0 kb and 23.1 kb, respectively; lane 3, uncut BAC DNA; lane 4, BAC DNA after digestion with *Bst*XI, with the largest band representing the desired 20.4 kb transgene; lane 5, the purified 20.4 kb *Bst*XI fragment used for injection, demonstrating relatively pure isolation of the desired band; and lane 6, the purified *Bst*XI fragment digested with *Eco*RI, yielding the expected DNA fingerprint. (C) The two-generation breeding scheme used to generate mice with the desired *nse5*/*nse5*, Tg(*Alg10b*) genotype (bottom right). Results of this cross are shown in Table 1.

To confirm that the *Alg10b* mutation in *nse5* animals is truly responsible for the hearing impairment phenotype, transgenic animals were generated that carry a 20.4 kb transgene derived from a bacterial artificial chromosome (BAC) containing a wildtype genomic copy of the *Alg10b* gene (Figures 3A & B). These animals were denoted +/+, Tg(*Alg10b*), to indicate wildtype alleles at the endogenous *nse5* locus and an additional wildtype *Alg10b* transgene array integrated elsewhere in the genome (Figure 3C). Of the 97 fertilized FVB eggs that were injected with the transgene DNA, 97 were transferred to surrogate mothers, and ten progeny animals were born. Of these ten mice, four carried the 129Sv allele of the *D15Jus1* locus within the transgene, indicating that the transgene had integrated into the genome of these four animals. (The wildtype *Alg10b* transgene was derived from the 129Sv mouse strain so that the transgene could be distinguished from the endogenous FVB *Alg10b* gene in transgenic animals, as well as from the mutant *Alg10b* gene, which is on a C57BL/6J background.)

These four animals were then crossed to *nse5*/*nse5* mutant animals, and the progeny that inherited the transgene, +/*nse5*, Tg(*Alg10b*) mice, were backcrossed to *nse5*/*nse5* mutant animals. The progeny of this cross are expected to be either heterozygous or homozygous for the *nse5* mutation at the endogenous *Alg10b* locus and to either have or lack the *Alg10b* transgene array, leading to four possible genotypes: (1) +/*nse5*; (2) *nse5*/*nse5*; (3) +/*nse5*, Tg(*Alg10b*); and (4) *nse5*/*nse5*, Tg(*Alg10b*) (Figure 3C). Animals with the *nse5*/*nse5*, Tg(*Alg10b*) genotype were expected to be hearing-impaired, unless the *Alg10b* transgene is capable of rescuing the mutant phenotype. Indeed, transgene rescue of the phenotype was seen in all four transgenic lines studied (Table 1), providing powerful evidence that the *Alg10b* mutation is responsible for the *nse5* mutant phenotype.

**Table 1:** Genotypes and Phenotypes of Animals from the *Alg10b* Transgene Rescue.

Transgenic Line Number	+/*nse5*	+/*nse5*, Tg(*Alg10b*)	*nse5*/*nse5*	*nse5*/*nse5*, Tg(*Alg10b*)
2	7	8	11	14
4	9	9	7	15
6	40	45	39	36
8	9	11	10	10
Phenotype	Hearing	Hearing	Deaf	Hearing

Phenotypes are based on click-box testing of animals, as described in the Materials and Methods.

### Auditory Brainstem Responses (ABRs), Distortion Product Otoacoustic Emissions (DPOAEs), and Cochlear Microphonics (CM) Confirm Hearing Impairment in Mutant Mice

**Figure 4:**
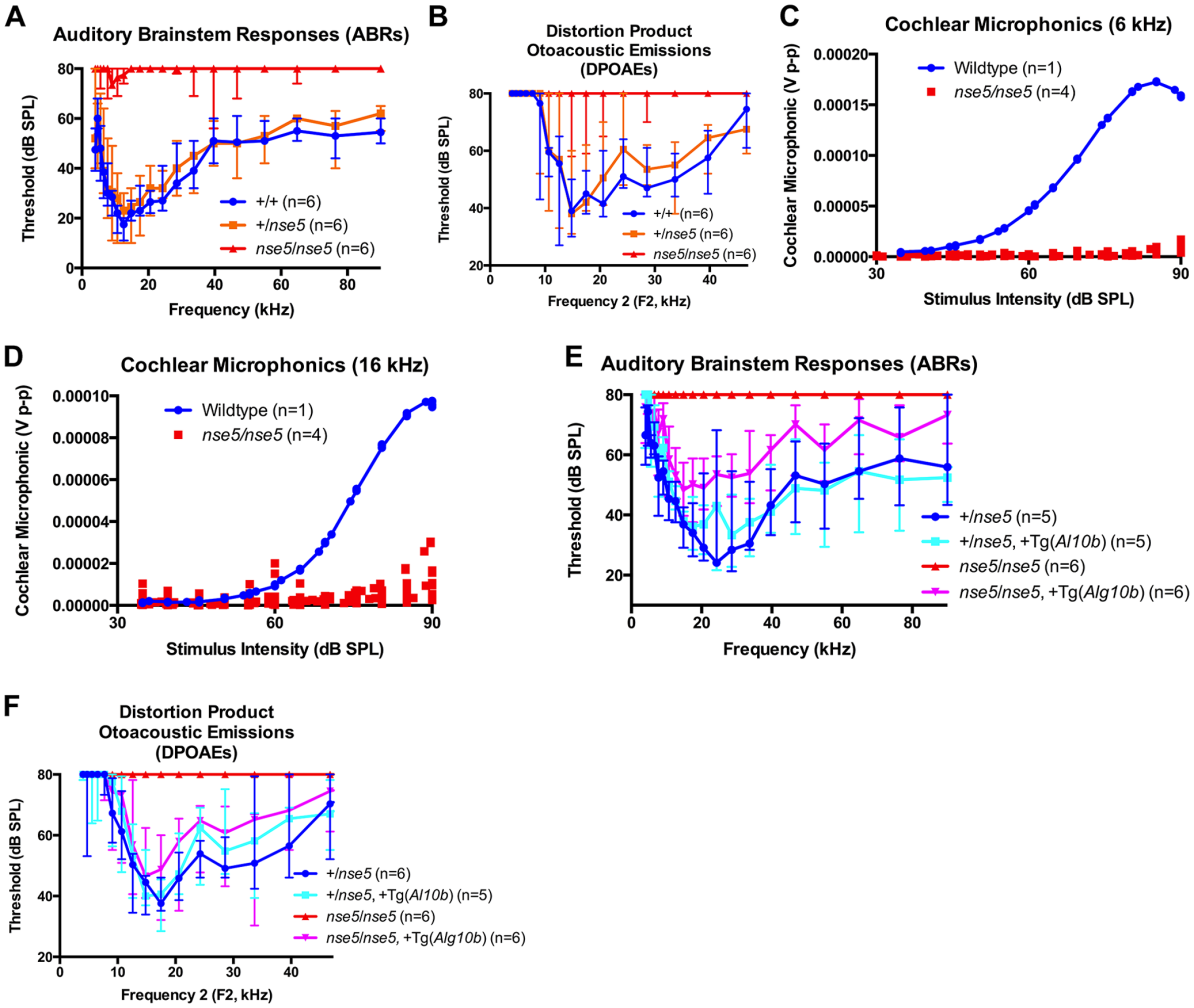
Auditory brainstem responses (ABRs), distortion product otoacoustic emissions (DPOAEs), and cochlear microphonics (CMs). (A) ABRs and (B) DPOAEs on six-week-old N10F1 mice. Each group consisted of 3 males and 3 females. Median values are plotted, with error bars representing the range of values for each group. The +/+ and +/*nse5* groups are indistinguishable, while the *nse5*/*nse5* group shows significant hearing impairment on both tests. CMs on four *nse5*/*nse5* animals reveal absent cochlear microphonics at both 6 kHz (C) and 16 kHz (D). ABRs (E) and DPOAEs (F) demonstrate at least a partial restoration of hearing in *nse5*/*nse5* mutant animals with the *Alg10b* wildtype transgene. db  =  decibels, SPL  =  sound pressure level, V p-p  =  voltage peak-to-peak.

ABRs and DPOAEs performed on six-week-old +/+, +/*nse5*, and *nse5*/*nse5* N10F1 animals revealed a significant hearing impairment in *nse5*/*nse5* homozygous animals. Heterozygous animals did not appear to be different from homozygous wildtype animals (Figures 4A & B). Endocochlear potential (EP) measurements performed on two +/+ and two *nse5*/*nse5* animals were virtually identical (93.3 milliVolts (mV) & 97.7 mV versus 102.2 mV & 101.1 mV, respectively), indicating normal ion concentrations in the perilymph versus the endolymph of the cochlea in mutant animals. By contrast, EPs are substantially reduced in mice homozygous for targeted *Gjb2* mutations in the cochlea [22]. CM measurements performed on four *nse5*/*nse5* homozygous mutant animals were found to be absent at both 6 kiloHertz (kHz) and 16 kHz (Figures 4 C & D). Taken together, these results suggest that the defect in *nse5*/*nse5* mutant animals involves the outer hair cells of the cochlea.

ABRs and DPOAEs performed on six-week-old animals from one of the four *Alg10b* transgenic lines (Line 6) confirmed transgene rescue of the *nse5* phenotype by the wildtype *Alg10b*, although the auditory brainstem responses did not completely normalize in transgenic animals at higher sound frequencies (Figures 4E & F).

### Immunohistochemistry Reveals Abnormalities in the Outer Hair Cells of *nse5* Animals

**Figure 5:**
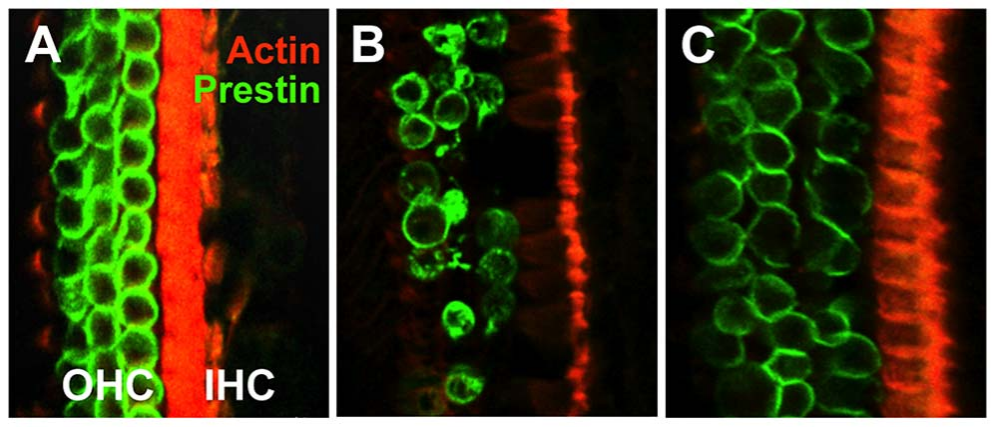
Immunohistochemistry reveals that the outer hair cells of the cochlea are abnormal in mutant animals. Cochleas were stained with phalloidin, which binds to filamentous actin (red) and anti-prestin antibody (green). In a two-month-old +/*nse5* heterozygote (A), the expected three rows of outer hair cells are seen on the left, stained green, and the one row of inner hair cells is seen on the right, with the stereocilia clearly visible and stained red. By contrast, the two-month-old *nse5*/*nse5* homozygote (B) shows a number of abnormalities, with numerous outer hair cells missing and punctate staining of prestin in many of the remaining cells. The stereocilia of inner hair cells, on the other hand, are still clearly visible in red on the right side of the image, though the phalloidin staining of the supporting cells between the inner and outer hair cells is substantially reduced. Examination of a 5½-month-old *nse5*/*nse5*, Tg(*Alg10b*) mouse (C) revealed preservation of the normal cochlear architecture, showing that the transgene rescue of the *nse5* mutant phenotype is long-lasting.

Immunohistochemistry was performed on inner and outer hair cells from cochleas of two-month-old heterozygous and homozygous mutant animals with phalloidin (which stains filamentous actin red) and anti-prestin antibody (which stains prestin green) (Figures 5A & B). Inner hair cells were relatively intact, based on the visualization of inner hair stereocilia in both heterozygous and homozygous mutant animals. Outer hair cells, on the other hand, appeared severely abnormal in homozygous mutant animals when compared to the heterozygous controls. Numerous outer hair cells were missing completely, while many others showed a punctate pattern of prestin staining in the cytosol, as opposed to the typical staining pattern showing prestin evenly associated with the cell membrane. This suggested an outer hair cell degenerative process was occurring. A similar analysis was performed on an older (5 ½ month-old) *Alg10b^nse5^* ^/*nse5*^, Tg(*Alg10b*) animal. Although the outer hair cells appeared swollen, they were all present and did not demonstrate evidence of intracellular prestin particles. This indicates that the transgene rescue of the mutant phenotype inhibits outer hair cell degeneration, preventing outer hair cell death over a relatively long time interval (Figure 5C).

## Discussion

Hearing impairment is one of the most common sensory impairments seen in the human population, and mouse models of hearing impairment have long been an avenue for the identification of human hearing impairment genes, as well as for understanding the physiology of hearing. Here, we report that a mutation in the gene for asparagine-linked glycosylation 10B (*Alg10b*) is responsible for the nonsyndromic hearing impairment observed in the *nse5* mouse strain. This was an unexpected finding, as the asparagine (N)-linked glycosylation pathway is highly evolutionarily conserved in eukaryotes, even in single-celled organisms such as yeast (*Saccharomyces cerevisiae*), where the pathway was first characterized, and it is estimated that thousands of human proteins undergo N-linked glycosylation [23]. Mutations in other human genes in this portion of the asparagine-linked glycosylation pathway are known to cause type I congenital disorders of glycosylation (CDGs). These are generally severe multisystem disorders with significant morbidity and mortality. Hearing impairment is sometimes, but not always, associated with these diseases [24]. However, unlike most of the other human genes in the N-linked glycosylation pathway, mutations in the human *ALG10B* gene have never been reported. A number of potential explanations could account for this failure.

First, *ALG10B* mutations in humans may cause nonsyndromic hearing impairment, as opposed to a multisystem disease, which would explain why no *ALG10B* mutations have previously been detected in a patient with a suspected CDG. However, an alternative explanation is that the *ALG10B* gene appears to be duplicated in humans (which have *ALG10A* and *ALG10B* genes, with the *ALG10A* gene sometimes being denoted as simply *ALG10* in the literature) but not in mice (which only have an *Alg10b* gene) [25]. Both copies of the human gene appear to be transcribed and spliced [19], and both are predicted to produce a full-length protein, though it is unclear if both proteins retain functional activity. Thus, it is possible that both copies of *ALG10A* and both copies of *ALG10B* would have to be mutated in humans to yield a phenotype.

*ALG10A* is located in the pericentric region of the short arm of human chromosome 12, whereas *ALG10B* is located in the pericentric region of the long arm of the same chromosome. Single chromosomal microdeletions involving both genes are extremely unlikely, as such a lesion would also delete the centromere of chromosome 12. However, it is possible that compound heterozygous mutations in both genes could lead to a phenotype, such as congenital hearing impairment or even age-related hearing impairment.

**Figure 6:**
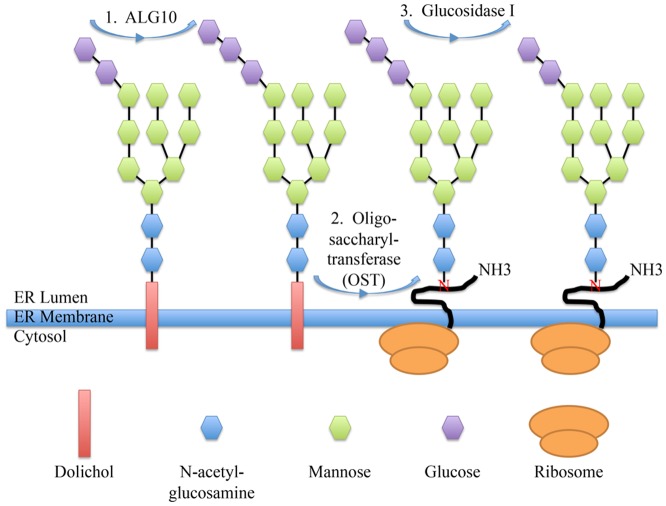
The role of ALG10B in Asparagine (N)-linked glycosylation. The ALG10B enzyme, initially characterized in yeast (shown here), catalyzes the transfer of the final glucose molecule to the glycan tree while it is still anchored to a dolichol molecule in the membrane of the endoplasmic reticulum (1). The entire glycan tree is then transferred to an asparagine residue on the target protein in the lumen of the endoplasmic reticulum (2), after which the terminal glucose is immediately cleaved off by glucosidase I (3). This step is believed to occur in order to prevent the glycan tree from being back-transferred to the dolichol molecule [47].

The link between N-linked glycosylation and nonsyndromic hearing impairment in the *nse5* mouse is quite intriguing. The enzymatic step catalyzed by the ALG10B protein during N-linked glycosylation is the addition of the last glucose molecule to the glycan “tree” inside the endoplasmic reticulum (ER), which is anchored to a dolichol molecule that is embedded in the membrane of the ER [26], [27]. After this step is completed, the glycan tree is transferred to an asparagine residue on the target protein by oligosaccharyltransferase (OST), and then the glucose molecule is immediately cleaved from the glycan tree by glucosidase I (encoded by the *CWH41* gene in yeast, the *Mogs* gene in mice (MGI: 1929872), and the *MOGS* gene in humans (OMIM *601336)) [23], [28] (Figure 6).

Given that the *Alg10b^nse5^* mutation is autosomal recessive, there are two possible explanations for why it is causing only nonsyndromic hearing impairment in mice, as opposed to a severe multisystem disorder. First, the *Alg10b^nse5^* mutation may be a hypomorphic mutation, and the mutant protein retains some residual activity. If this is the case, then the outer hair cells of the cochlea are exquisitely sensitive to a reduction in the function of the ALG10B protein, while other cells in the body are less sensitive and require less ALG10B function to maintain sufficient N-linked glycosylation of key proteins. Alternatively, the *Alg10b^nse5^* mutation may represent a null allele, in which case the ALG10B protein is required for the normal functioning of the outer hair cells but is dispensable in all other cell types.

Needless to say, yeast is not traditionally thought of as a model organism for hearing impairment, but the bulk of the research on *ALG10B* has been carried out in yeast. *ALG10B* was discovered in yeast almost two decades ago, and its function was rapidly elucidated [26], [27]. However, deletion of the *ALG10B* gene in yeast does not completely abolish N-linked glycosylation, suggesting that there is some redundancy in the pathway [27]. The *ALG6*, *ALG8*, and *ALG10B* genes all appear to share a common ancestor, which could explain this redundancy [29]. Furthermore, deletion of the *ALG10B* gene in yeast does not result in a detectable growth phenotype [27]. Indeed, the only phenotype that has been found in *ALG10B* yeast mutants is “a round cell shape and a partial unipolar distal budding pattern” in diploid yeast, suggesting a possible role in establishing and maintaining cell polarity [30].

Intriguingly, the only multicellular species for which a mutation in the *ALG10B* gene could be found prior to our studies is a plant—*Arabidopsis thaliana*. Mutant plants grow normally in Murashige and Skoog (MS) medium, but they have smaller leaves than normal when grown in soil, and root growth is hypersensitive to salt concentration relative to controls [31].

How, then, do we explain the disparate phenotypes between yeast, *Arabidopsis*, and mice with mutations in the gene for ALG10B? We propose that, as in yeast, the ALG10B protein has functional overlap with the ALG6 and ALG8 proteins in *Arabidopsis* and mice, so that mutations in the gene for ALG10B in both species reduce the efficiency of N-linked glycosylation rather than eliminating it completely. Under this hypothesis, a small subset of proteins is extremely sensitive to perturbations in the N-linked glycosylation pathway, whereas most proteins are tolerant of the defect and can function normally. This subset of ultrasensitive proteins would be very different in *Arabidopsis* versus mice, which accounts for the differences in mutant phenotypes. Identification of this subset of proteins in mice, which may include prestin itself, will be a rich area of study for future experiments involving the role of N-linked glycosylation in development and maintenance of normal hearing.

## Materials and Methods

### Mice

The *nse5* mutation arose in the progeny of an inbred, ENU-treated C57BL/6J mouse and was previously mapped to mouse chromosome 15 [18]. Mice carrying the C57BL/6J alleles of *D15Mit192* and *D15Mit241* were backcrossed to inbred 129S6/SvEvTac mice for at least ten generations (N10) to generate a congenic mouse strain for further studies. Animals heterozygous for the C57BL/6J alleles of *D15Mit192* and *D15Mit241* from the N10 and N11 generations were intercrossed to produce N10F1 and N11F1 animals, which were used to fine-map the mutation and the characterize the mutant phenotype.

Tg(*Alg10b*) mice were generated by pronuclear injection into FVB embryos in the Baylor College of Medicine (BCM) Genetically Engineered Mouse (GEM) core, as described below.

### Ethics Statement

All animals were housed in the Transgenic Mouse Facility at Baylor College of Medicine, a specific pathogen free (SPF) mouse facility that is accredited by the Association for Assessment and Accreditation of Laboratory Animal Care (AAALAC). Animals were provided food and water *ad libitum*. All work was approved by the Institutional Animal Care and Use Committee (IACUC) at Baylor College of Medicine.

### Initial Phenotyping and Fine-Mapping of the *nse5* Mutation

All N10F1 and N11F1 animals were genotyped at the *D15Mit192* and *D15Mit241* loci and phenotyped as either “Hearing” or “Deaf” based on the presence or absence of a “Preyer” reflex when presented with a 90 decibel (dB) sound pressure level (SPL) 20 kiloHertz (kHz) sound burst. This was performed using custom-built click-boxes obtained from Dr. Karen P. Steel. The “Preyer” reflex is a startle response consisting of backward-flicking of the ear pinnae and occasionally whole-body flinching by a hearing animal [32], [33].

Animals with a recombination breakpoint between *D15Mit192* and *D15Mit241* were identified by discordance between the genotypes at these two loci. Recombinant animals were then genotyped at the *rs33858577* and *D15Mit171* loci to further localize the recombination breakpoint. Animals with recombination events between *rs33858577* and *D15Mit171* were then genotyped at the *rs33858727* locus, while animals with recombination events between *D15Mit171* and *D15Mit241* were genotyped at the *rs6260777* locus. Animals carrying critical recombinants (i.e., between *D15Mit171* and *rs6260777*) were then crossed to *nse5*/*nse5* animals, and at least five animals that inherited the recombinant chromosome were phenotyped to deduce the genotype at the *nse5* locus, in order to guard against the possibility of incorrectly phenotyping an animal with a critical recombinant.

### Polymerase Chain Reaction (PCR)

DNA was isolated from key animals via a tail biopsy as described [34]. The concentration of each sample was determined via spectrophotometry, and then each sample was diluted to a concentration of 25 ng/µl with Tris-EDTA (TE) buffer [35].

PCR primers for the *D15Mit171*, *D15Mit192*, and *D15Mit241* markers have been previously published [36]. Primers for all other amplicons were designed using the Primer3 program [37] and are shown in Table 2. The first exon of *Alg10b* was extremely difficult to amplify and was ultimately obtained with the GC-RICH PCR System (Roche Applied Science, Indianapolis, IN) with the following PCR cocktail: 50 ng (2 µl) genomic DNA, 12.5 pmol forward and reverse primers, 5 nmol dNTPs, 2.5 µl GC-RICH Resolution Solution, 5 µl 5X GC-RICH Reaction Buffer, and 0.5 µl GC-RICH Enzyme Mix, in a total volume of 25 µl. The resulting product was gel purified, reamplified, and gel-purified again prior to sequencing. All other PCR reactions consisted of 50 ng (2 µl) genomic DNA, 12.5 pmol forward and reverse primers, 5 nmol dNTPs, 2.5 units *Taq* DNA polymerase, and 1X Standard *Taq* Buffer (New England Biolabs, Ipswich, MA) in a total volume of 25 µl. For PCR products less than 500 bp, the thermocyler profile was: 94 degrees for 3 minutes; 30 cycles of 94 degrees for 30 seconds, 55 degrees for 30 seconds, and 72 degrees for 30 seconds; 72 degrees for 10 minutes; and 4 degrees until the products were ready for analysis. For PCR products longer than 500 bp, the extension time was increased to 90 seconds. Appropriate control DNAs from parental inbred strains were included in every reaction set.

**Table 2:** PCR and Sequencing Primers.

Primer Name	Sequence
*rs33858577*.for	5′-GTC TAA GCC AGT CCC TGT GC-3′
*rs33858577*.rev	5′-ACA GAC AGG TGC TGA TGC TG-3′
*rs33858727*.for	5′-AGC ACT AGG GGG TTC TGG TT-3′
*rs33858727*.rev	5′-TTT TCC CGC CTC TCT TCA TA-3′
*rs6260777*.for	5′-CAA AGA ATC AAT AAA GAG CTG GTT C-3′
*rs6260777*.rev	5′-TTG GTT TTC CAA CGA GTC AA-3′
*Alg10b*_exon_1.pcr.for	5′-CGA AGG GCT GGT TGT ATG TT-3′
*Alg10b*_exon_1.pcr.rev	5′-GGC CCT AAG ATC CTC CAC TC-3′
*Alg10b*_exon_1.seq.for	5′-CTT CCG TCC GAC GCT CTC-3′
*Alg10b*_exon_1.seq.rev	5′-CAC TAG GCA GCT CCC TTC AC-3′
*Alg10b*_exon_2.for	5′-TGG GAT AAC TCT TGC TGT GC-3′
*Alg10b*_exon_2.rev	5′-AGG TTT GAA AAA CAG ACA CCA-3′
*Alg10b*_exon_3.for	5′-GGT GTT GCA AAG CCA GAA AT-3′
*Alg10b*_exon_3.rev	5′-CAC GAC AAC TGC AAG CAA CT-3′
*D15Jus1*.for	5′-FAM-TGG TGA AAT AAG TGC ATT GAC A-3′
*D15Jus1*.rev	5′-GTG TGA ATT TCC ACG GAG GT-3′

### Genotyping

The *D15Mit171*, *D15Mit192*, and *D15Mit241* markers were all scored visually by subjecting the PCR products to gel electrophoresis on a 4% MetaPhor agarose (Lonza Rockland, Rockland, ME), 0.5X TBE gel run at 100V. PCR products representing *rs33858577* were digested with *Eco*RI and then scored visually after gel electrophoresis on a standard 2% agarose, 0.5X TBE gel run at 100V [35]. The *rs33858727* and *rs6260777* markers were analyzed by PCR followed by dideoxynucleotide sequencing, as described below. The *D15Jus1* marker was analyzed by running the products on ABI 3730*xl* DNA Analyzer with the GeneScan-1000 ROX Size Standard (Applied Biosystems, Carlsbad, CA) or the MapMarker 1000 X-Rhodamine ladder (BioVentures, Inc., Murfreesboro, TN) and analyzing the data with the Peak Scanner software program (Applied Biosystems, Carlsbad, CA).

### Dideoxynucleotide Sequencing

PCR products were either sent directly to Agencourt Biosciences (Beverly, MA) for purification and dideoxynucleotide suquencing or were gel-purified with the DNA Clean & Concentrator-5 kit (Zymo Research, Orange, CA), run through the BigDye Terminator v3.1 Cycle Sequencing kit (Applied Biosystems, Carlsbad, CA), followed by another purification with CENTRI-SEP columns (Princeton Separations, Adelphia, NJ), and then sent to either the Molecular Core Laboratory at Baylor College of Medicine (Houston, TX) or GENEWIZ (South Plainfield, NJ) to be run on a Genetic Analyzer. With the exception of exon 1 of *Alg10b* (as noted in Table 2), all products were sequenced with the same primers that were used for the initial PCR. The results were visualized with the Sequencher software program (Gene Codes, Ann Arbor, MI).

### Generation and Breeding of *Alg10b* Transgenic Animals

The bMQ bacterial artificial chromosome (BAC) library is a genome-wide, end-sequenced BAC library derived from the 129S7/SvEvBrd-*Hprt* ^b-m2^ mouse strain [38]. Based on the published end-sequence data, the 78.4 kb BAC bMQ33j10 was determined to contain a complete genomic copy of the *Alg10b* gene. Bacteria containing this BAC were obtained and cultured overnight in 500 ml Luria broth with 20 µg/ml chloramphenicol in a 37 degree Celsius air shaker. High-quality BAC DNA was isolated with the NucleoBond BAC 100 kit (Clontech Laboratories, Inc., Mountain View, CA), per the manufacturer’s instructions, with minor modifications. (For the “Clarification of the lysate step”, the filtration option was used, and the subsequent filtrate was *not* centrifuged prior to adding it to the column.) The DNA was resuspended in 180 µl ddH2O. A restriction digestion was set up as follows: 135 µl BAC bMQ33j10 DNA, 15 µl 10X buffer “O”, and 15 µl 10 U/ul *Bst*XI (Fermentas, Inc., Glen Burnie, MD). This reaction was incubated at 55 degrees Celsius for three hours to completely digest the BAC DNA. 15 µl of loading dye was then added to the reaction, and all of it was run on a 0.8% agarose, 1X TAE gel at 100 V for six hours. The running buffer was changed at the three-hour mark. The large 20.4 kb band was excised from the gel, and the DNA was extracted from this gel slice with the QIAEXII Gel Extraction Kit (QIAGEN, Inc., Valencia, CA), per the manufacturer’s instructions. The DNA was eluted into 60 µl of injection buffer [34] and the mixture was spun at 16,100 x g for 15 minutes to remove any particulate matter from the suspension prior to injection into fertilized eggs. The DNA was estimated to be at a concentration of 12 ng/µl based on gel electrophoresis. 10 µl of DNA was then combined with 290 µl of injection buffer for a final concentration of 0.4 ng/µl, and this mixture was injected into 97 fertilized FVB mouse eggs [34].

### *In vivo* Experiments

Procedures performed on anesthetized mice included the measurement of ABRs, DPOAEs, EPs, and CMs. Mice were anesthetized using ketamine (100 mg/kg) and xylazine (5 mg/kg). Supplemental doses of anesthesia were administered to maintain areflexia to paw pinch. All methods have been fully described elsewhere [39]–[41] but are described briefly below.

Sine wave stimuli were generated digitally using MATLAB (Release 13, The Mathworks, Natick, MA), converted to analog signals using a digital-to-analog converter running at 200 kiloHertz (kHz), and then attenuated to the appropriate intensity according to our experimental design (Part numbers RP2 and PA5, Tucker-Davis Technologies, Alachua, FL) [42]. To generate the acoustic stimuli, high frequency piezoelectric speakers were used (EC1, Tucker-Davis Technologies). The speakers were connected to an ear bar inserted into the ear canal and calibrated from 4 to 95 kHz by a probe-tip microphone (type 8192, NEXUS conditioning amplifier, Bruel and Kjar, Denmark) inserted through the ear bar. The tip of the microphone was within 3 millimeters of the tympanic membrane.

The ABR signal was measured with a bioamplifier (Part number DB4, Tucker-Davis Technologies) from a needle electrode positioned at the ventral surface of the tympanic bulla referenced to an electrode placed at the vertex of the skull, as previously described [43], [44]. A ground electrode was placed in the hind leg. The stimulus was a 5 millisecond (ms) sine wave tone pip of alternating polarity with cos^2^ envelope rise and fall times of 0.5 ms and a repetition time of 50 ms. The stimulus intensity ranged from 10 to 80 decibels (dB) SPL (sound pressure level) in 10 dB steps. The frequency range studied was 4 to 90 kHz. Two hundred and fifty ABR responses were sampled at 25 kHz over the 50 ms repetition time and averaged. Thresholds were calculated off-line. At each frequency, the peak-to-peak voltage of the ABR waveform was measured and the data interpolated over the range of stimulus intensities. The ABR threshold was determined at four standard deviations above the noise floor. If no ABR response was detected even at our equipment limits of 80 dB SPL, we arbitrarily defined the threshold to be 80 dB SPL.

DPOAE thresholds were measured as previously described [41]. Briefly, the stimuli for eliciting DPOAEs were two sine wave tones of differing frequencies (F1 and F2; F2 = 1.2*F1) of one-second duration with F2 ranging from 4 to 90 kHz. The two tones were presented at identical intensities, which ranged from 20 to 80 dB SPL in 10 dB increments. The acoustic signal detected by the microphone in the ear bar was digitized at 200 kHz and the magnitude of the 2*F1-F2 distortion product determined by FFT (Fast Fourier Transform). The surrounding noise floor was also calculated by averaging 20 adjacent frequency bins around the distortion product frequency. DPOAE thresholds were calculated off-line by interpolating the data and identifying when the signal was >-5 dB SPL and greater than two standard deviations above the noise floor. If no DPOAE response was detected even at our equipment limits of 80 dB SPL, we arbitrarily defined the threshold to be 80 dB SPL.

For EP measurements, the tympanic bulla was opened, and the bone overlying the basal turn was thinned with a microknife very close to the stapedial artery to expose the spiral ligament. A glass micropipette pulled to a tip diameter of about 1 micrometer filled with 150 millimolar KCl was advanced up to the spiral ligament with a micromanipulator. The voltage was measured every 1 second (s) referenced to a silver wire placed under the skin near the vertex of the skull (Axoclamp-2, Axon Instruments). The baseline bias voltage was compensated for by determining the zero-current potential of the extra-cochlear fluids outside of the spiral ligament, before entering scala media. After entering the endolymphatic space, the EP was recorded until the measurement stabilized (usually about 5–10 s). Then, the micropipette was withdrawn from the cochlea and the voltage of the extra-cochlear fluids re-measured to verify that no baseline drift occurred.

The CM is a field potential that reflects the summation of hair cell transduction currents primarily from outer hair cells at the basal turn of the cochlea. After rigidly securing the mouse in a head holder, the pinna was surgically resected. The bulla was carefully opened medial to the tympanic annulus to expose the round window. The stapedial artery was preserved. The ear bar was then inserted into the ear canal and secured. The CM was measured from the ball-ended tip of a Teflon-coated silver wire (0.003 inch diameter, A-M Systems, Carlsborg, WA) advanced onto the round window membrane with a micromanipulator. The signal was referenced to a silver wire inserted under the skin near the vertex of the skull. The ground electrode was placed in the hind leg. A bioamplifier was used (Part number DB4, Tucker Davis Technologies) to amplify the signals 100 times, and no filtering was used. The stimulus was a 30 ms 6 kHz tone, repeated every 1 s, with an intensity range from 10–100 dB SPL. By measuring the speaker output with the probe tip microphone in the ear bar, FFT analysis demonstrated that all stimulus harmonics and noise at all other frequencies were at least 50 dB below the primary signal at all stimulus intensities. The CM signal measured by the bioamplifier was digitized at 200 kHz, and the magnitude of the response at 6 kHz and 16 kHz determined by FFT.

### Whole-mount preparations to view the hair cell epithelium

Mice were anesthetized and sacrificed by cervical dislocation. Their cochleae were removed and immersed in a 313±2 mOsm/kg artificial perilymph solution containing 150 mM NaCl, 5 mM KCl, 1.5 mM CaCl_2_, 10 mM HEPES, and 10 mM glucose at pH 7.35. Under a dissecting microscope (SteREO Discover.V8, Zeiss, Germany), the vestibular structures and ossicles were carefully removed. The cochleae were then fixed in 4% paraformaldehyde at room temperature for two hours and then glued upright into custom-built chambers. Once the cochleae were secured, the otic capsule over the basal turn was removed with a fine knife. The outer hair cell epithelium was exposed after Reissner’s membrane and the tympanic membrane were removed with a pick.

The cochleae were permeabilized with 0.1% Triton X-100 in PBS and simultaneously stained with Alexa Fluor 568 Phallodin (Invitrogen; 1∶200 in phosphate-buffered solution) for 15 minutes at room temperature. Immunolabeling was performed by first blocking the organs of Corti with 4% donkey serum (017-000-121, Jackson Immuno Research Laboratories, West Grove, PA) in PBST for 1 hour at room temperature and then incubating with the primary antibody at 4°C overnight. Specimens were washed three times with PBST and then incubated with the secondary antibody at room temperature for 1 hour. The primary antibody was goat anti-prestin N-20 (1∶500; SC-22692, Santa Cruz Biotechnology, Santa Cruz, CA), and the secondary antibody was Alexa Fluor 488 donkey anti-goat (1∶500; Invitrogen, Grand Island, NY).

Labeled cochleae were imaged using a custom-built two-photon microscope [45]. Briefly, the microscope consisted of a moveable objective microscope (MOM; Sutter) fitted with a 20x water-immersion objective (NA 0.95, XLUMPlanFl, Olympus America, Center Valley, PA). A femtosecond Ti:sapphire laser (Chameleon, Coherent, Santa Clara, CA) tuned to 800 nanometers (nm) provided two-photon excitation. The emitted fluorescence was detected by two photomultiplier tubes after optical filtering. Lateral scanning was achieved by two galvanometer-actuated mirrors controlling the laser beam. Axial scanning was achieved by a separate actuator that controlled the objective lens. A modified version of ScanImage open source software [46] was used to control the hardware.

## References

[1] Gaffney M, Eichwald J, Grosse SD (2010) Identifying infants with hearing loss - United States, 1999–2007. MMWR Morb Mortal Wkly Rep 59: 220–223. 20203554

[2] Petersen MB, Willems PJ (2006) Non-syndromic, autosomal-recessive deafness. Clin Genet 69: 371–392. 16650073 10.1111/j.1399-0004.2006.00613.x

[3] Ross SA, Novak Z, Kumbla RA, Zhang K, Fowler KB, et al (2007) GJB2 and GJB6 mutations in children with congenital cytomegalovirus infection. Pediatr Res 61: 687–691. 17426645 10.1203/pdr.0b013e3180536609

[4] Raviv D, Dror AA, Avraham KB (2010) Hearing loss: a common disorder caused by many rare alleles. Ann N Y Acad Sci 1214: 168–179. 21175685 10.1111/j.1749-6632.2010.05868.x PMC3689008

[5] Lenz DR, Avraham KB (2011) Hereditary hearing loss: from human mutation to mechanism. Hear Res 281: 3–10. 21664957 10.1016/j.heares.2011.05.021

[6] Gregg RB, Wiorek LS, Arvedson JC (2004) Pediatric audiology: a review. Pediatr Rev 25: 224–234. 15231988 10.1542/pir.25-7-224

[7] Izumikawa M, Minoda R, Kawamoto K, Abrashkin KA, Swiderski DL, et al (2005) Auditory hair cell replacement and hearing improvement by Atoh1 gene therapy in deaf mammals. Nat Med 11: 271–276. 15711559 10.1038/nm1193

[8] Raphael Y, Frisancho JC, Roessler BJ (1996) Adenoviral-mediated gene transfer into guinea pig cochlear cells in vivo. Neurosci Lett 207: 137–141. 8731440 10.1016/0304-3940(96)12499-x

[9] Akil O, Seal RP, Burke K, Wang C, Alemi A, et al (2012) Restoration of hearing in the VGLUT3 knockout mouse using virally mediated gene therapy. Neuron 75: 283–293. 22841313 10.1016/j.neuron.2012.05.019 PMC3408581

[10] Lentz JJ, Jodelka FM, Hinrich AJ, McCaffrey KE, Farris HE, et al (2013) Rescue of hearing and vestibular function by antisense oligonucleotides in a mouse model of human deafness. Nat Med 19: 345–350. 23380860 10.1038/nm.3106 PMC3657744

[11] Probst FJ, Camper SA (1999) The role of mouse mutants in the identification of human hereditary hearing loss genes. Hear Res 130: 1–6. 10320095 10.1016/s0378-5955(98)00231-7

[12] Leibovici M, Safieddine S, Petit C (2008) Mouse models for human hereditary deafness. Curr Top Dev Biol 84: 385–429. 19186249 10.1016/S0070-2153(08)00608-X

[13] Brown SD, Hardisty-Hughes RE, Mburu P (2008) Quiet as a mouse: dissecting the molecular and genetic basis of hearing. Nat Rev Genet 9: 277–290. 18283275 10.1038/nrg2309

[14] Vrijens K, Van Laer L, Van Camp G (2008) Human hereditary hearing impairment: mouse models can help to solve the puzzle. Hum Genet 124: 325–348. 18784944 10.1007/s00439-008-0556-y

[15] Hardisty RE, Mburu P, Brown SD (1999) ENU mutagenesis and the search for deafness genes. Br J Audiol 33: 279–283. 10890141 10.3109/03005369909090110

[16] Bosman EA, Penn AC, Ambrose JC, Kettleborough R, Stemple DL, et al (2005) Multiple mutations in mouse Chd7 provide models for CHARGE syndrome. Hum Mol Genet 14: 3463–3476. 16207732 10.1093/hmg/ddi375

[17] Vissers LE, van Ravenswaaij CM, Admiraal R, Hurst JA, de Vries BB, et al (2004) Mutations in a new member of the chromodomain gene family cause CHARGE syndrome. Nat Genet 36: 955–957. 15300250 10.1038/ng1407

[18] Kile BT, Hentges KE, Clark AT, Nakamura H, Salinger AP, et al (2003) Functional genetic analysis of mouse chromosome 11. Nature 425: 81–86. 12955145 10.1038/nature01865

[19] Kent WJ, Sugnet CW, Furey TS, Roskin KM, Pringle TH, et al (2002) The human genome browser at UCSC. Genome Res 12: 996–1006. 12045153 10.1101/gr.229102 PMC186604

[20] Mouse Genome Sequencing C, Waterston RH, Lindblad-Toh K, Birney E, Rogers J, et al (2002) Initial sequencing and comparative analysis of the mouse genome. Nature 420: 520–562. 12466850 10.1038/nature01262

[21] Adzhubei IA, Schmidt S, Peshkin L, Ramensky VE, Gerasimova A, et al (2010) A method and server for predicting damaging missense mutations. Nat Methods 7: 248–249. 20354512 10.1038/nmeth0410-248 PMC2855889

[22] Crispino G, Di Pasquale G, Scimemi P, Rodriguez L, Galindo Ramirez F, et al (2011) BAAV mediated GJB2 gene transfer restores gap junction coupling in cochlear organotypic cultures from deaf Cx26Sox10Cre mice. PLoS One 6: e23279. 21876744 10.1371/journal.pone.0023279 PMC3158073

[23] Schwarz F, Aebi M (2011) Mechanisms and principles of N-linked protein glycosylation. Curr Opin Struct Biol 21: 576–582. 21978957 10.1016/j.sbi.2011.08.005

[24] Freeze HH, Aebi M (2005) Altered glycan structures: the molecular basis of congenital disorders of glycosylation. Curr Opin Struct Biol 15: 490–498. 16154350 10.1016/j.sbi.2005.08.010

[25] Scherer SE, Muzny DM, Buhay CJ, Chen R, Cree A, et al (2006) The finished DNA sequence of human chromosome 12. Nature 440: 346–351. 16541075 10.1038/nature04569

[26] Zufferey R, Knauer R, Burda P, Stagljar I, te Heesen S, et al (1995) STT3, a highly conserved protein required for yeast oligosaccharyl transferase activity in vivo. EMBO J 14: 4949–4960. 7588624 10.1002/j.1460-2075.1995.tb00178.x PMC394598

[27] Burda P, Aebi M (1998) The ALG10 locus of Saccharomyces cerevisiae encodes the alpha-1,2 glucosyltransferase of the endoplasmic reticulum: the terminal glucose of the lipid-linked oligosaccharide is required for efficient N-linked glycosylation. Glycobiology 8: 455–462. 9597543 10.1093/glycob/8.5.455

[28] Romero PA, Dijkgraaf GJ, Shahinian S, Herscovics A, Bussey H (1997) The yeast CWH41 gene encodes glucosidase I. Glycobiology. 7: 997–1004. 10.1093/glycob/7.7.997 9363442

[29] Oriol R, Martinez-Duncker I, Chantret I, Mollicone R, Codogno P (2002) Common origin and evolution of glycosyltransferases using Dol-P-monosaccharides as donor substrate. Mol Biol Evol 19: 1451–1463. 12200473 10.1093/oxfordjournals.molbev.a004208

[30] Ni L, Snyder M (2001) A genomic study of the bipolar bud site selection pattern in Saccharomyces cerevisiae. Mol Biol Cell 12: 2147–2170. 11452010 10.1091/mbc.12.7.2147 PMC55669

[31] Farid A, Pabst M, Schoberer J, Altmann F, Glossl J, et al (2011) Arabidopsis thaliana alpha1,2-glucosyltransferase (ALG10) is required for efficient N-glycosylation and leaf growth. Plant J 68: 314–325. 21707802 10.1111/j.1365-313X.2011.04688.x PMC3204403

[32] Steel KP, Hardisty R (1996) Assessing hearing, vision and balance in mice. Society of Neuroscience Short Course Syllabus No 1: What's wrong with my mouse? Washington DC. pp. 26–38.

[33] Kiernan AE, Zalzman M, Fuchs H, Hrabe de Angelis M, Balling R, et al (1999) Tailchaser (Tlc): a new mouse mutation affecting hair bundle differentiation and hair cell survival. J Neurocytol 28: 969–985. 10900098 10.1023/a:1007090626294

[34] Nagy A, Gertsenstein M, Vintersten K, Behringer R (2003) Manipulating the mouse embryo: A laboratory manual, 3rd ed. Cold Spring HarborNY: Cold Spring Harbor Press. 764 p.

[35] Sambrook J, Russell DW (2001) Molecular cloning: A laboratory manual, 3rd ed. Cold Spring Harbor, NY: Cold Spring Harbor Laboratory Press.

[36] Copeland NG, Jenkins NA, Gilbert DJ, Eppig JT, Maltais LJ, et al (1993) A genetic linkage map of the mouse: current applications and future prospects. Science 262: 57–66. 8211130 10.1126/science.8211130

[37] Rozen S, Skaletsky HJ (2000) Primer3 on the WWW for general users and for biologist programmers. In: Krawetz S, Misener S, editors. Bioinformatics Methods and Protocols: Methods in Molecular Biology. Totowa, NJ: Humana Press. pp. 365–386. 10.1385/1-59259-192-2:365 10547847

[38] Adams DJ, Quail MA, Cox T, van der Weyden L, Gorick BD, et al (2005) A genome-wide, end-sequenced 129Sv BAC library resource for targeting vector construction. Genomics 86: 753–758. 16257172 10.1016/j.ygeno.2005.08.003

[39] Liu CC, Gao SS, Yuan T, Steele C, Puria S, et al (2011) Biophysical mechanisms underlying outer hair cell loss associated with a shortened tectorial membrane. J Assoc Res Otolaryngol 12: 577–594. 21567249 10.1007/s10162-011-0269-0 PMC3173552

[40] Xia A, Gao SS, Yuan T, Osborn A, Bress A, et al (2010) Deficient forward transduction and enhanced reverse transduction in the alpha tectorin C1509G human hearing loss mutation. Dis Model Mech 3: 209–223. 20142329 10.1242/dmm.004135 PMC2869304

[41] Xia A, Visosky AM, Cho JH, Tsai MJ, Pereira FA, et al (2007) Altered traveling wave propagation and reduced endocochlear potential associated with cochlear dysplasia in the BETA2/NeuroD1 null mouse. J Assoc Res Otolaryngol 8: 447–463. 17701252 10.1007/s10162-007-0092-9 PMC2538339

[42] Oghalai JS (2004) Chlorpromazine inhibits cochlear function in guinea pigs. Hear Res 198: 59–68. 15567603 10.1016/j.heares.2004.03.013

[43] Wenzel GI, Anvari B, Mazhar A, Pikkula B, Oghalai JS (2007) Laser-induced collagen remodeling and deposition within the basilar membrane of the mouse cochlea. J Biomed Opt 12: 021007. 17477714 10.1117/1.2714286 PMC3651902

[44] Wenzel GI, Xia A, Funk E, Evans MB, Palmer DJ, et al (2007) Helper-dependent adenovirus-mediated gene transfer into the adult mouse cochlea. Otol Neurotol 28: 1100–1108. 18043435 10.1097/MAO.0b013e318158973f

[45] Yuan T, Gao S, Saggau P, Oghalai J (2010) Calcium imaging of inner ear hair cells within the cochlear epithelium of mice using two-photon microscopy. J Biomed Opt 15: 016002. 20210449 10.1117/1.3290799 PMC2821419

[46] Pologruto TA, Sabatini BL, Svoboda K (2003) ScanImage: flexible software for operating laser scanning microscopes. Biomed Eng Online 2: 13. 12801419 10.1186/1475-925X-2-13 PMC161784

[47] Helenius A, Aebi M (2004) Roles of N-linked glycans in the endoplasmic reticulum. Annu Rev Biochem 73: 1019–1049. 15189166 10.1146/annurev.biochem.73.011303.073752

